# The Need of the Hour

**Published:** 1890-08

**Authors:** 


### THE NEED OF THE HOUR.

THERE are periods in human activity, as there are cycles in all progress, in which we have the slow movement upward, the rapid rotation, and the dead centre of stagnation.

The profession of dentistry has passed through these various stages in common with all active human effort. The present seems to be one of unusual activity in certain directions, and the earnest work infuses new life into all departments of labor. In proportion to the increase in mental effort there follows the critical period,—the unrest,—the grasping for the ideal,—the, perhaps, unattainable.

This thought finds its origin in the variety of topics discussed in the dental journals, outside of purely professional matters. At times these have bordered very closely on hypercriticism, forcing the conclusion that, possibly, a personal feeling has influenced the writers; or in others, perhaps, the true scientific interest, in building up weak lines, is paramount. This is unquestionably the true evidence of progress. The idea that advancement can be made on a union of sentiment has long been discarded by those able to analyze, even to a limited degree, the forces which actuate mental states. We do not need so much a unification of thought as we do harmony in effort. The notes of discord may be present everywhere, but they are only the signs of the revolution of ideas towards a common centre.

It seems to be a mistake that some regard with disfavor any efforts except such as coincide with their ideas. The new college, the new journal, the new machine are all alike distasteful to the conservative mind; nevertheless the forces that are constantly resolving the elemental into the concrete are not bounded by such limitations of thought.

Dentistry is no exception to the general law. The men of today are not where their fathers were, or should not be. The old days of isolation have passed by, and the ruts of the ancient roads of scientific knowledge alone remain to remind us of the immaturity of the early experiences.

Are we building better than the fathers? Are we sowing the seeds of criticism to germinate into the flower, fair to the eye but poisonous to contact? Do we forget that the parts of every machine differ in size, form, and function; but all serve to unity of effort?

We are led to these remarks by noticing a tendency to fault-finding permeating the writings of many. It is the association here, the college there, and the journal always. The difficulties that surround all these forms of professional effort are numerous, and rarely understood by the uninitiated. The critics should go to deeper soundings. They should remember that evolution has not yet made the perfect man, possibly never will, and until better men are developed all things will remain imperfect.

If such be the crudity of professional work, what is the duty of each member of this calling of ours? If we are on the ascending scale, are we not in danger of future stagnation? This may certainly come; but it will meet us more quickly if each one say to himself, "I will let some one else do the work, 'but as for me and my house,' we will serve self." How many of the twenty thousand dentists in this country are to-day reasoning from this lower standard? Count the number actively engaged in professional work, men and women who attend conventions, are active in their local societies, who contribute their quota to the journals, through their local, State, or national organizations, and it will be found that the remainder constitutes a heavy drag to progress.

There is work to do for every man and woman engaged in the practice of dentistry to-day, not a work that will build a fine house, give you an elegant span of horses, clothe yourself and family in the best apparel; or that which will tend only to narrowing your efforts to individual gratification. Let these broaden in a professional outlook, and do something worthy of the calling of which you are an integral part.

The conventions, national and international, have closed their work for this year. The men who "compassed sea and land" to make these worthy of this professional period, did they do this for their own selfish gratification? The question needs no answer. They with you were worn with the labors of the year; but you will be found either delving at home for gold, at the risk of a broken constitution, or you are seeking mountain air and sea-shore with the remark, it may be, "Oh, I never trouble dental conventions!" Is this the right spirit? We need more and more the self-sacrificing feeling that will lay down a portion of all that we may have upon the altar of professional worship.

We are reminded that it will be said, "as the world has been so will it always be;" but we prefer to believe that the world will be as we design to make it, and we, each of us, are responsible whether it be a pandemonium or a paradise; whether professionally it means from this time an inferior body of workers or an ever-increasing standard of excellence. Which shall it be?

